# Impact of YouTube® videos on knowledge on tracheal intubation for anesthesiologist trainees: a prospective observational study

**DOI:** 10.1186/s44158-025-00232-3

**Published:** 2025-02-25

**Authors:** Annachiara Marra, Andrea Uriel de Siena, Carmine Iacovazzo, Maria Vargas, Nicola Cesarano, Claudia Collà Ruvolo, Giuseppe Celentano, Pasquale Buonanno

**Affiliations:** 1 Department of Neurosciences, Reproductive and Odontostomatological Sciences, University of Naples “ Federico II”, Naples, Italy; 2 Department of Neurosciences, Reproductive and Odontostomatological Sciences, Urology Unit, University of Naples Federico II, Naples, Italy

**Keywords:** YouTube®, Tracheal intubation, Education, Video, Learning

## Abstract

**Background:**

Social media platforms, initially developed for recreational use, have evolved into major sources for disseminating information, including medical information for patients and healthcare providers in many disciplines. This study aimed to evaluate the educational potential of YouTube® videos in teaching tracheal intubation to first-year anesthesiology residents.

**Methods:**

This study was approved by the Ethical Committee of “University Federico II-AORN A. Cardarelli” (protocol no. 00010735). We analyzed the first 10 YouTube videos identified via the search term “tracheal intubation.” Each video was evaluated for misinformation and informational completeness via a 5-item Likert scale. After providing written consent, fifty-seven first-year anesthesiology residents watched these videos and completed a 22-item questionnaire before and after viewing. Each correct answer received one point, whereas incorrect answers received 0 points, allowing for comparisons of knowledge acquisition.

**Results:**

The videos exhibited moderate quality (median score, 3; range, 1–5) and low informational completeness (median score, 1.432; range, 1.000–2.046). Residents’ scores increased modestly by one point after viewing (from 13 to 14; *p* < 0.001). Misinformation was positively correlated with the number of followers (beta coefficient: 0.00002, *p* < 0.001), video duration (beta coefficient: 0.0042, *p* < 0.05), and linking ratio (beta coefficient: 0.242, *p* < 0.05). Conversely, informational completeness was inversely correlated with video duration (beta coefficient: − 0.001121, *p* < 0.05) and the thumbs-up/view ratio (beta coefficient: − 67.4697, *p* < 0.05).

**Conclusions:**

While YouTube® has potential as an accessible educational tool, current video selection offers limited improvement in residents’ understanding of tracheal intubation. Our findings highlight the need for greater curation and better-quality control of medical educational content on YouTube® to optimize its effectiveness and provide accurate information. Institutions could play a key role in producing reliable, guideline-based videos that better support learning objectives in anesthesiology training.

## Introduction

Social media has become central in modern life, with increasing consultation because their vast and unexplored potential continues to emerge [1]. While various attempts have been made to define social media, all definitions converge on the central theme of information sharing via web-based platforms, creating a dynamic internet community marked by two-way communication [2]. Social media can be categorized into six distinct types: (1) collaborative projects, such as Wikipedia; (2) blogs, microblogs, and internet forums (i.e., Wordpress.com); (3) content communities, such as YouTube® for videos; (4) social networking sites such as Facebook, Ning, LinkedIn, and MySpace; (5) virtual game worlds (i.e., the World of Warcraft); and 6) virtual social worlds, such as Second Life [3]. Among content communities, YouTube stands out as the most widely used, with over one billion videos watched daily, 30 million daily active users, and 500 h of video uploaded every minute. This platform has become a powerful tool for making video content accessible globally to a vast audience. However, like other social media, YouTube® lacks filters or mechanisms to verify the accuracy of information, posing a risk of misinformation, especially for less-informed users [4]. Although primarily utilized for recreational purposes, YouTube hosts a significant volume of educational content, including medical information geared toward both patients and healthcare professionals.

The use of YouTube® for medical education was explored by some systematic reviews and metanalysis and other original articles in various fields [5–9]. A systematic review highlighted that social media tools can effectively supplement traditional medical training, offering interactive and accessible educational experiences. Specifically, the development of concise online videos, such as the mini-GEMs series in geriatric medicine, has demonstrated the value of targeted e-learning resources in improving knowledge retention and clinical skills [6]. Moreover, surveys among surgical professionals indicate a growing reliance on streaming platforms like YouTube® for procedural learning and guideline dissemination, underscoring the platform’s role in modern medical education [5, 9]. These findings suggest that YouTube could be used as an additional toolkit for anesthesiology residents, providing readily accessible content that can enhance traditional learning modalities; nevertheless, a low level of knowledge of medicine may not be sufficient to evaluate the quality of information on highly specialized topics. This is especially relevant for residents who are not yet experts in their field, such as trainees. Residents may turn to YouTube for practical and easily accessible demonstrations that are difficult to convey through textbooks, such as hands-on procedures that benefit from visual representation [10, 11].

The purpose of this study was to investigate the educational potential of YouTubeTM videos for tracheal intubation, specifically by evaluating the quality of the information presented and assessing their effectiveness in enhancing the knowledge of anesthesiology residents.

## Materials and methods

We performed a search on YouTube on 10 September 2024 at 10:00 p.m. using the term “tracheal intubation” after logging out of the personal account and setting up a virtual private network (VPN) in the USA. We selected videos in English with automatically generated Italian subtitles faithful to the original language. Videos with no explanations of the procedure performed on pediatric patients and procedures performed on COVID-19 patients were excluded. We included only videos with durations ranging from 2 to 15 min in which the intubation was performed through both direct and video laryngoscopy. We selected the first 10 videos resulting from the YouTubeTM search, which met the abovementioned criteria.

After completion of the video research, each video was independently analyzed by two authors (P.B. and N.C.); possible disagreements were solved by discussion or through the intervention of a third author (A.M.). We recorded all the following characteristics of the selected videos: length in minutes, total views, views per month, thumbs up (defined as like), thumbs down (defined as dislike), likes per view, dislike per view, number of comments, and number of followers. After collecting the data, we evaluated the popularity and impact of each video by calculating the like ratio (such as *100/[like + dislike]), the view ratio (number of views/days), and the video power index (such as the like ratio*view ratio/100) (VPI); like ratio, view ratio, and VPI are scores used for the first time by Erdem et al. to assess the popularity of a video and thereafter used by many other authors investigating the impact of YouTube content on the viewers [12–15].

We created a 22-item questionnaire in the Italian language (Supplemental Materials) divided into three domains: 9 questions about what to do before tracheal intubation, 11 about what to do during the procedure, and 3 about what to do immediately after. We assigned 1 point for each correct answer and 0 points for each wrong answer to calculate the score reported by the residents.

The videos were first analyzed to assess the misinformation level via a 5-item Likert scale (1-no; 2-very little; 3-moderate; 4-high; 5-extreme misinformation). In our analysis, the term misinformation referred not to completeness but to the quality and accuracy of the information in the videos. The completeness of information was evaluated with a 5-item Likert scale on the basis of the percentage of questions the single video answered: 1–0–19%; 2–20–39%; 3–40–59%; 4–60–79%; and 80–100%. The score was recorded for all three domains of the questionnaire (before, during, and after intubation), and the overall score was calculated as a weighted mean of the three domains.

The inclusion criterion for the students was an anesthesiologist who was a resident in the first year of the degree and who provided formal written consent. The exclusion criteria were residents who left the school before the start of the study, residents who did not provide written consent, and residents who joined the school after the start of the study.

After ethical approval, the data collection started. After providing written informed consent, the residents were gathered in a classroom to watch the videos; they were asked to complete the questionnaire immediately before and immediately after watching the videos. We enrolled all 57 residents attending the first year of our school of specialization in anesthesiology (University of Naples “Federico II”) before they started their training in the operating room in November 2024.

### Statistical analysis

The distribution of the data was explored by the Shapiro‒Wilk test; the parametric data are presented as the means and standard deviations, and the nonparametric data are presented as the medians and ranges. Paired data were analyzed through Student’s *t* test for paired data if normally distributed, whereas paired nonparametric data were analyzed through the Wilcoxon signed-rank test for matched data. Kendall–Thiel regression was performed to investigate the correlations between variables. The results were considered statistically significant when *p* < 0.05. All analyses were performed via R studio and its packages “mblm” and “rstatix” [16–19]. The total sample size was calculated via G*Power with a Wilcox signed-rank test for matched pairs with two-tails, an effect size of 0.45, an alpha error of 0.05, and a beta error of 0.2, resulting in 43 students [20].

## Results

There were 57 enrolled students.

Table 1 shows the main characteristics of the selected videos. The median length was 288 min (range: 124–839). The median number of views and views per month were 118,240 (range: 1883–3,704,001) and 3059 (range: 47–53,219), respectively. The median number of thumbs up, thumbs down, thumbs up per view, and thumbs down per view were 917 (range: 23–31,407), 38 (range: 0–1178), 0.0085 (range: 0.0024–0.0137), and 0.000252 (range: 0–0.000618), respectively. The median numbers of comments and followers were 42 (range: 1–416) and 8065 (range: 451–224,000), respectively, whereas the median length of stay on YouTube® was 46.6 months (range: 15.5–126.9). The median like ratio, view ratio, and VPI were 97.12 (93.22–100), 101.965 (1.574–1773.983), and 98.28 (1.57–1721.72), respectively. The median completeness scores before, during, and after intubation were 1 (range: 1–1), 1.5 (range: 1–3), and 1 (range: 1–4), respectively; the median overall score for completeness of information was 1.432 (range: 1.000–2.046).

**Table 1:** Characteristics of YouTube® videos

**Parameter**	**Result**
**YouTube® metric**	
Madian length of video, min (range)	288 (124–839)
Median of views ratio, *n* (range)	101.965 (1.574–1773.983)
Median views/month	3059 (47–53219)
Median thumbs up	917 (23–31407)
Median thumbs down	38 (0–1178)
Median thumbs up/views	0.0085 (0.0024–0.0137)
Median thumbs down/views	0.000252 (0–0.000618)
Median number of comments, *n* (range)	42 (1–416)
Median followers, *n* (range)	8065 (451–224000)
Median duration on YouTube®, months (range)	46.6 (15.5–126.9)
Like ratio, *n* (range)	97.12 (93.22–100)
VPI, *n* (range)	98.28 (1.57–1721.72)
**Source of upload**	
Universities/scientific societies, *n*	1
Private practice/non-academic hospital or institution	8
Commercial	1
**Speaker**	
Health care providers	7
External voice	3
**Intended audience**	
Health care providers	9
Patients	1
**Misinformation**	3 (1–5)
**Completeness of information**	
Before	1 (1–1)
During	1.5 (1–3)
After	1 (1–4)
Overall	1.432 (1.000–2.046)

*VPI* video power index

Table 2 shows the scores recorded before and after watching the videos: the total score improved from 13 to 14 (*p* < 0.0001); the analysis of the subsets of scores regarding questions about what to do before, during, and after intubation revealed that only the score during intubation significantly increased (*p* < 0.001).

**Table 2:** Median score before and after watching videos

**Questions**	**Before**	**After**	***P* value**
Total, median (range)	13 (2–19)	14 (3–20)	< 0.0001
Before Intubation, median (range)	5 (1–8)	5 (1–8)	0.6031
During Intubation, median (range)	6 (0–10)	7 (1–10)	< 0.001
After Intubation, median (range)	2 (0–3)	2 (0–3)	< 0.0001

Table 3 reports the regression analysis. Regression analysis revealed that misinformation is directly proportional to followers (beta coefficient: 0.00002, *p* < 0.01), the duration of the videos (beta coefficient: 0.0042,* p* < 0.05), and the like ratio (beta coefficient: 0.242, *p* < 0.05) (Table 3).

**Table 3:** Regression analysis for misinformation

**Variable**	**Estimate**	***P* value**
Views	1.394e − 07	0.79985
View ratio	0.0000000	0.55340
Followers	2.013e − 05	0.00903
Thumbs up	1.653e − 05	0.79985
Thumbs down	0.000000	1.00000
Duration	0.004252	0.0243
Days on YouTube®	0.0000000	0.2807
Comments	0.001337	0.35254
Completeness of information		
Total	0.0000	0.7536
Pre		
During	− 0.5000	02355
Post	− 0.0416	0.83784
Like ratio	0.2416	0.036
VPI	0.0000	0.55340

*VPI* video power index

The completeness of information was found to be inversely correlated with duration (beta coefficient: − 0.001121, *p* < 0.05) and thumbs up/views (beta coefficient: − 67.4697, *p* < 0.05) (Table 4).

**Table 4:** Regression analysis for completeness of information

**Variable**	**Estimate**	***P* value**
Views	− 1.320e − 07	0.19213
Views/months	− 9.163e − 06	0.19213
Followers	− 2.394e − 06	0.12288
Thumbs up	− 1.519e − 05	0.34283
Thumbs down	− 0.0003817	0.55327
Duration	− 0.001121	0.02427
Days on YouTube®	0.0001927	0.15477
Comments	− 0.002748	0.05248
Like ratio	− 0.04458	0.141
View ratio	− 0.0002749	0.19213
VPI	− 0.0002849	0.19213
Thumbs up/views	− 67.4697	0.03282
Thumbs down/views	− 1615.0931	0.05736

*VPI* video power index

## Discussion

Our study aimed to evaluate the educational value of YouTube videos on tracheal intubation for anesthesiology residents. Although the residents’ scores significantly improved after watching the selected videos, this improvement was modest and cannot be deemed educationally meaningful. Notably, misinformation was directly correlated with the number of followers, video duration, and the like ratio, whereas informational completeness was inversely correlated with duration and the thumbs-up/view ratios. To our knowledge, this is the first study to directly assess the educational impact of YouTube videos on residents. While previous studies have evaluated the accuracy of YouTube medical content aimed at physicians or patients, no prior research has examined the platform’s effectiveness in transferring correct information to learners [4, 10, 21–24]. YouTube® usage has grown significantly, with 64.2% of the global population having internet access by 2020 [2]. One key reason for its popularity among medical students is the rapid and convenient access to the information it offers [25]. However, concerns have been raised about the reliability of online medical content, as there is little to no source verification, allowing any user to post material freely [8]. Prior research has suggested that YouTube videos generally demonstrate moderate quality in terms of educational value for students [4, 26]. Our findings align with this finding, revealing a moderate level of misinformation in the selected videos. Notably, misinformation was directly correlated with the number of followers, video duration, and video-like ratio, suggesting that a high subscriber count does not guarantee informational accuracy. More concerningly, this finding indicates that viewers may lack the ability to critically evaluate content quality. Misinformation was also inversely related to informational completeness, while misinformation reflects content accuracy, informational completeness addresses the breadth of information (i.e., the number of questions answered from our questionnaire). Additionally, we observed that longer videos provided more comprehensive information but also conveyed greater misinformation.

Residents’ scores slightly but statistically significantly improved after watching the videos. However, the median score increased by only one point, indicating minimal educational value. The improvement was mostly limited to the procedural aspects of intubation rather than the pre- and postintubation phases. This suggests that video content is heavily focused on the procedure itself, with limited attention given to assessing difficult intubation predictors or verifying tube placement afterward.

YouTube® could serve as a valuable educational resource for anesthesiology residents, especially in areas such as tracheal intubation where visual learning is beneficial. Anesthesiology requires both a solid theoretical foundation and hands-on proficiency in various procedures, with tracheal intubation being a critical skill. In this context, the available videos reinforce a common misconception among residents—that intubation is simply a technical skill of placing a tube in the trachea; instead, emphasis should be placed on preventing and managing potential complications which represent the real challenge of every anesthesiology procedure.

This study has several limitations. First, we used a non-validated questionnaire to assess residents’ knowledge acquisition, and the scales for misinformation and informational completeness were also invalidated. Second, our sample size was restricted to first-year residents in our anesthesiology program; a larger cohort might yield more robust results. Third, we could not verify the identity or medical background of YouTube users who provided likes or dislikes on videos, meaning that the like ratio may not reflect feedback from a medically educated audience. Finally, YouTube® search results vary across users and are influenced by factors such as previous search history, geographical location, language, and current platform trends.

## Conclusion

In conclusion, our study suggests that YouTube could be a potential educational tool for anesthesiology residents, but alarmingly, the most popular videos are those characterized by a higher risk of misinformation. We believe that institutions, including universities and international medical societies, should actively engage in this emerging educational arena by producing videos that provide accurate information aligned with current clinical guidelines.

## Data Availability

The datasets used and/or analysed during the current study are available from the corresponding author on reasonable request.
